# Method and phantom to study combined effects of in‐plane (x,y) and z‐axis resolution for 3D CT imaging

**DOI:** 10.1120/jacmp.v17i5.6294

**Published:** 2016-09-08

**Authors:** David Goodenough, Josh Levy, Smari Kristinsson, Jesper Fredriksson, Hildur Olafsdottir, Austin Healy

**Affiliations:** ^1^ Department of Radiology The George Washington University Washington DC USA; ^2^ The Institute For Radiological Image Sciences Myersville MD USA; ^3^ The Phantom Laboratory Salem NY USA; ^4^ Image Owl Salem NY USA; ^5^ Raförninn Reykjavik Iceland

**Keywords:** CT, 3D imaging, slice thickness, MTF, Fourier analysis

## Abstract

Increasingly, the advent of multislice CT scanners, volume CT scanners, and total body spiral acquisition modes has led to the use of Multi Planar Reconstruction and 3D datasets. In considering 3D resolution properties of a CT system it is important to note that both the in‐plane (x,y) and z‐axis (slice thickness) influence the visualization and detection of objects within the scanned volume. This study investigates ways to consider both the in‐plane resolution and the z‐axis resolution in a single phantom wherein analytic or visualized analysis can yield information on these combined effects. A new phantom called the “Wave Phantom” is developed that can be used to sample the 3D resolution properties of a CT image, including in–plane (x,y) and z‐axis information. The key development in this Wave Phantom is the incorporation of a z‐axis aspect of a more traditional step (bar) resolution gauge phantom. The phantom can be examined visually wherein a cutoff level may be seen; and/or the analytic analysis of the various characteristics of the waveform profile by including amplitude, frequency, and slope (rate of climb) of the peaks, can be extracted from the Wave Pattern using mathematical analysis such as the Fourier transform. The combined effect of changes in in‐plane resolution and z‐axis (thickness), are shown, as well as the effect of changes in either in‐plane resolution, or z‐axis thickness. Examples of visual images of the Wave pattern as well as the analytic characteristics of the various harmonics of a periodic Wave pattern resulting from changes in resolution filter and/or slice thickness, and position in the field of view are shown. The Wave Phantom offers a promising way to investigate 3D resolution results from combined effect of in‐plane (x‐y) and z‐axis resolution as contrasted to the use of simple 2D resolution gauges that need to be used with separate measures of z‐axis dependency, such as angled ramps. It offers both a visual pattern as well as a pattern amenable to analytic analysis using Fourier Transform methods, and is believed to offer an image quality test closer to the diagnostic task where the 2D image has the hidden third (z) axis effects.

PACS number(s): 87.57.Q‐

## I. INTRODUCTION

Classical measures of CT resolution generally involve separate measures of in‐plane (x,y) resolution and slice thickness (z‐axis). In fact, the actual clinical image is produced by the separate measures acting together in tandem.^(1)^

The ability of the tomographic imaging device to produce accurate images, including 3‐dimensional renditions of objects, may be vital in applications involving diagnosis of disease, volume measurements, and three‐dimensional planning of invasive medical procedures.^(2)^

The aim of this work is to use a newly patented phantom that can be used to simultaneously sample the x, y (or radial) and third‐dimension (z‐axis) extent of a tomographic image rather than making separate measurements of in–plane (x, y) or z‐axis (thickness) information.^(3)^ Phantoms are desired that can not only produce a visual pattern to aid in the evaluation of the radial and three‐dimensional properties of the image, as for example the Lucy 3D QA Phantom,^(4)^ but also are amenable to mathematical analysis of the x‐, y‐, and z‐axis image properties separately as well as in a simultaneous fashion. An embodiment of the Wave Ramp was recently incorporated into a commercially available CT phantom (Catphan 700, The Phantom Laboratory, Salem, NY).^(5)^

**Figure 1:**
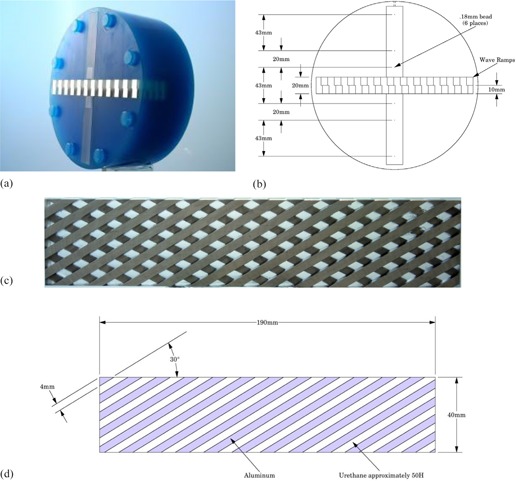
Wave phantom: (a) photo; (b) top view (x,y plane) of 20 cm phantom with dimensions of components; (c) side view of pair of angled ramps; (d) schematic figure, showing side view of one of the ramp angulations, with dimensions. The composition of this ramp is alternating spacing of Aluminum and a tissue‐similar plastic.

The Wave Phantom is illustrated in Fig. 1 and is designed as a stand‐alone module test object, insertable into the Catphan CT Phantom, and can be used as a single test object of 3D resolution, rather than requiring separate test objects for in‐plane (x,y) and z‐axis resolution.^(6)^

## II. MATERIALS AND METHODS

### A. Theoretical aspects

Our approach uses a phantom as illustrated in Fig. 1. The phantom utilizes a periodic pattern embedded as a module of a pair of angled ramps (30°), configured to produce a waveform profile of pixel values of the CT image that results from scanning the pair of angled ramps.^(2)^ The trigonometric effect of the angle is accounted by the scaling for the magnified ramp size in the x‐y plane and is applied, as appropriate, to scale the Z dimensions of the ramps being used. A pair of ramps with opposed angles is used to provide two separate measurements, as well as a visual and quantitative sense of scan angle alignment, by noting any differences in ramp pattern dimensions that will tend to expand or diminish as the relative ramp angle would be changed by gantry and/or phantom angulation.

**Figure 2:**
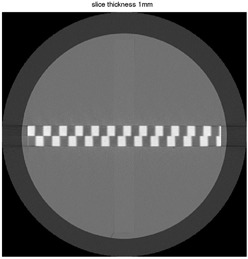
CT scan of the Wave Pattern taken on a Toshiba Aquilion One CT scanner with a 1 mm slice thickness and a medium‐high resolution kernel.

When the test object is imaged, the axial CT image of the test object will show a waveform profile that comprises a pattern of repeating on and off signal values of, as seen in Fig. 2.

A perfect image with near zero loss of resolution in the x‐, y‐, z‐planes of a tomographic slice of the test object would produce a consistent pattern in the square wave waveform profile taken across a set of angled ramps in the image, due to the consistent parallel spacing of the angled ramps, as well as the consistent thickness of each angled ramp. However, due to the finite resolution in the spatial performance of a tomographic imaging device, the CT waveform profile across the set of angled ramps will not yield the perfect waveform profile. The actual profile will be influenced by spatial resolution limitations caused by the finite z‐axis slice thickness and x‐y in‐plane resolution, as well as stochastic (noise) limitations and any other sources of nonuniformity such as beam hardening, faulty calibration of the CT scanner, and geometric mismatch (such as angulation) of the object with the scan plane.

Various characteristics of the waveform profile including, but not limited to, amplitude, frequency and slope (rate of climb) of the peaks, as well as associated mathematical analyses of the waveform profile, such as the Fourier transform, can be analyzed to determine and evaluate imaging performance of the tomographic imaging device. Spatial performance includes the device's ability to accurately image an object, including in‐plane resolution characteristics, slice thickness, angular orientation of the slice plane, and uniformity of response across the scan field. If, for instance, there is a variation in slice thickness throughout the slice or from one side of the slice to another, or if the mean of the profile is changing due to nonuniformity of the scanner, these variations will be reflected in the image of the test object and the waveform profiles taken across the angled ramps in the image will encode these properties.

The waveform profiles from the tomographic images may be visually examined which in some circumstance may be sufficient to provide a general evaluation of the performance of the imaging device. Alternatively, or additionally, the waveform profiles may be mathematically analyzed — for instance, using automatic processing software.

These concepts will now be examined theoretically as a function of slice thickness and in‐plane resolution before examining experimental results in the following section.

### B. Effect of slice thickness (z‐axis) on ramp profile

**Figure 3:**
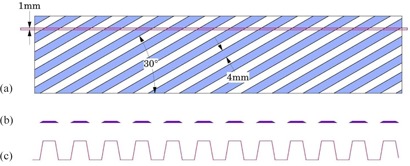
Schematic illustrations of the wave phantom: (a) side view of intercept of a 1 mm slice with one of the angled ramps; (b) intercept intensity pattern; (c) waveform.

To further illustrate some of the concepts of how a waveform profile can encode characteristics of a tomographic imaging device reference is made to Fig. 3. In Fig. 3(a) the side view of the interception of an ideal (uniform) 1 mm tomographic slice with the test object is depicted; in Fig. 3(b) the intercept pattern is shown; the corresponding wave profile is shown in Fig. 3(c). The thickness of the slice is vertical in this illustration and would correspond to the z‐axis in conventional CT scanning. A given slice intercepts portions of angled ramps separated by cast material. The resulting image represents only those portions that are present in this particular slice.

**Figure 4:**
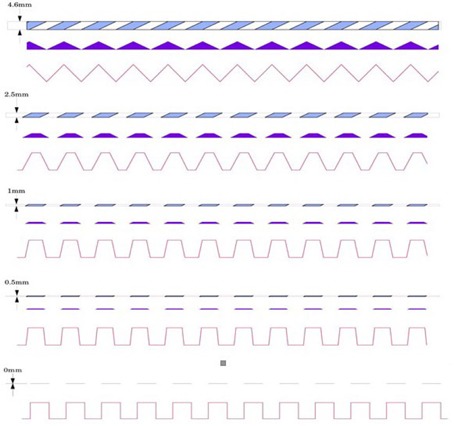
Waveform profile characteristics with varying slice thickness ranging from 0.0 mm to 4.6 mm.

Likewise, Fig. 4 depicts similar slice and waveform profile characteristics as those depicted in Fig. 3, but with ideal uniform slice thickness, varying from 0.0 mm to 4.6 mm.

Figure 4 further illustrates that, as slice thickness decreases towards an infinitely thin slice, the shape of the corresponding waveform would approach a formal “square wave”, with infinitely steep slopes and flat peaks.

**Figure 5:**
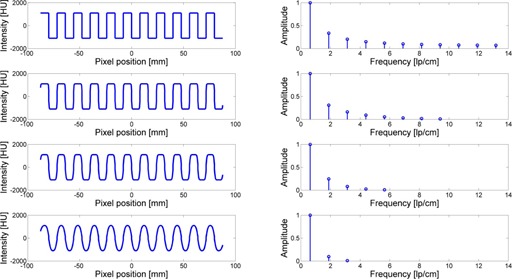
Profiles (left); harmonics (right). Top to bottom: no smoothing, smoothing with a Gaussian kernel using sigma = 0.5, 1, and 2. In all cases, pixel resolution is kept constant at 0.5 mm.

It is well known that an (infinite) square wave pattern is characterized by the well‐known Fourier series and resulting harmonics, as provided from Eq. (1) and as shown in Fig. 5 (top):

(1) $$\begin{array}{rl} & f_{\text{square}}(x)=\frac{4}{\pi }\sum_{k=1}^{\infty }\frac{sin(2\pi (2k–1)vx)}{\left(2k–1\right)}\\ & =\frac{4}{\pi }\left(sin(2\pi vx)+\frac{1}{3}sin(6\pi vx)+\frac{1}{5}sin(10\pi vx)+\cdots \right), \end{array}$$

where x is the spatial distance and *v* is the spatial frequency and the weighing factors on the contributing sine waves constitute the harmonics of the function and decreases as: 1/1; 1/3; 1/5; … etc.^7^, ^8^, ^9^

However, a waveform plot extracted from images obtained by a tomographic imaging device will exhibit a rounding and/or blurring characteristic of the profile resulting in a repetitive more sine wave‐like pattern. As mentioned previously, the two major reasons are (i) the influence of finite z‐slice thickness and (ii) in‐plane (x‐y) point‐spread function (psf), or blur resolution limitations in the x‐y plane of the imaging device.^10^, ^11^

The effects of changes of in‐plane (x‐y) resolution will show similar changes in wave form plots of Fig. 4 if z‐axis slice thickness had been kept constant but in‐plane resolution varied. For instance, as illustrated in Fig. 5, as x‐y resolution decreases, the psf increases in size (blurring increases) and the waveform plot will become more rounded and exhibit less extreme slopes in the peaks/valleys of the waveform, much like the effects of increased slice thickness, but with continuous effects across the plane, not just at the intercepted region of the slice thickness with the angled ramps.

## III. RESULTS & ANALYSIS

### A. Comparison with measures of in‐plane resolution vs. 3D wave resolution

**Figure 6:**
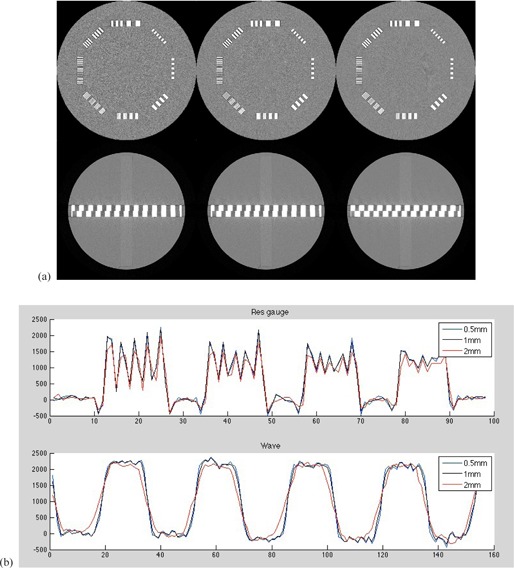
Comparable scans (a) of Catphan 700 resolution gauge section (top) vs. the Catphan 700 Wave Pattern scans (bottom) on a Toshiba Aquilion One scanner at varying slice thicknesses: left to right 0.5 mm, 1 mm, and 2 mm. Profiles (b) through the 12−15lp/cm section of the Catphan 700 Phantom from the 0.5, 1.0, and 2 mm slices all showing modulation through 14lp/cm compared to the profiles through the Wave Phantom at corresponding slice thickness where the profiles are shown to broaden as the slice thickness increases. Several adjacent pixel rows are averaged to reduce the effect of statistical noise.

To try to illustrate some of the attributes of the Wave Phantom, and particularly differences from existing methods of measuring resolution, such as two‐dimensional (2D) resolution gauges, Fig. 6(a) shows a comparable scan of traditional resolution gauges as used in the Catphan 700 CT Phantom. These patterns range from 1 to 30 lp/cm.

In this case, a medium–high resolution kernel was used to scan the phantom on a Toshiba Aquilion One Scanner (Toshiba, Tokyo, Japan) at slice thicknesses of 0.5 mm, 1.0 mm, and 2 mm. Of course, since the reconstruction filter is constant, the only difference in the resolution gauges should be the increased statistical noise from the lower slice thicknesses. In contradistinction, the Wave Pattern with the added variation in the third (z‐axis) dimension shows differences in the periodic pattern variation. This is the key qualitative difference in a resolution pattern that has 3D variation as opposed to the more classical 2D resolution patterns.

As seen in the scans in Fig. 6(a) of the 2D resolution gauge vs. the 3D Wave Pattern gauge, and as mentioned in the previous theoretical section, the waveform profiles taken across an image of a tomographic slice may be mathematically analyzed to evaluate spatial performance of the imaging device. For example, a Fourier Transform (FT) is a decomposition of a signal into component frequencies, as utilized for instance in the previously mentioned Fourier Series of infinite square wave pattern, or for example when using a Modulation Transfer Function (MTF).^(10)^ In imaging systems, the mathematical properties of Eq. (1) have been used to characterize the relationship between the contrast transfer function of square wave pattern compared to the sinusoidal varying patterns of the MTF.^(12)^

Thus, for example in Fig. 6(b) one can see the waveform profiles of CT numbers in the 12 to 15lp/cm section of the 2D resolution gauges vs. the 3D Wave Pattern for three different slice thicknesses (0.5 mm, 1 mm, 2 mm) all taken with the same resolution kernel (medium‐high).

**Figure 7:**
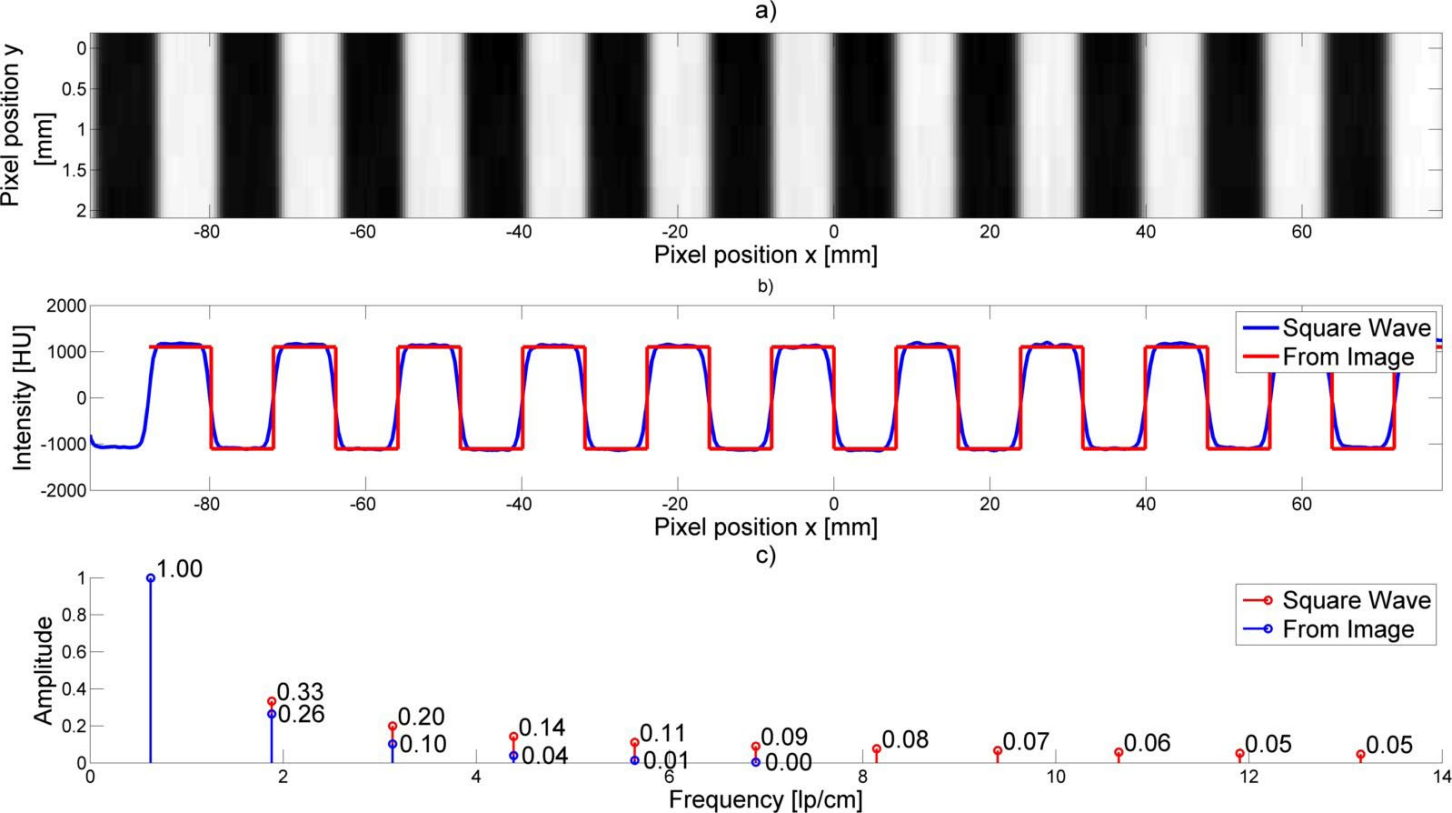
A cropped waveform (top) from a tomographic slice. The extracted intensity profile (middle) compared to an ideal square wave pattern. The corresponding Fourier transform (bottom) of both profiles including the theoretical amplitude (upper) and measured experimental amplitudes (lower).

To illustrate the further analytic use of the Wave Phantom, Fig. 7 provides a visual example of the wave fluctuations and resulting harmonics of the Fourier transform of the Wave Phantom. The waveform profile reveals the clear presence and amplitude of first, second, and third harmonics. The harmonics of the actual CT profile reflect not only the in‐plane 2D resolution effects on the harmonics of the theoretical amplitudes of a perfect square wave because the periodic pattern is “rounded” by the level of in‐plane resolution, but also by the slice thickness effects in the z‐axis, further reducing the harmonic amplitudes. The extent of the reduction in amplitude should reflect the overall modulation transfer function (MTF) at the frequency of interest for each harmonic. The overall MTF will reflect the combination of the normal in‐plane MTF, as well as the additional reduction due to the MTF of the slice thickness profile.

### B. Effect of changing slice thickness

**Figure 8:**
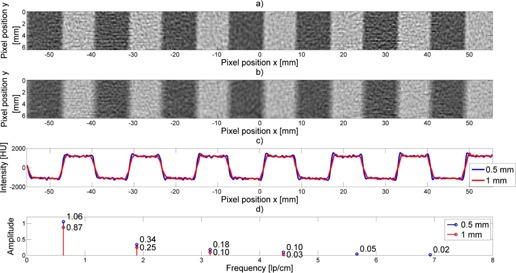
Effect of changing slice thickness. Waveform from (a) 0.5 mm and (b) 1 mm thick CT slice. A superimposition (c) of the extracted waveform profiles. The resulting amplitudes (d) of the Fourier harmonics for each profile. Note, the amplitude of the 0.5 mm first harmonic exceeds 1, probably reflecting the increased MTF from a 0.5 mm slice and/or use of a small focal spot, resulting in some edge enhancement as seen in (c).

Figure 8 depicts two other waveform profiles and their corresponding Fourier Transforms from a thicker slice (1 mm) and thinner (0.5 mm) slice when using the same in‐plane resolution. As can be seen, Fourier transform of the waveform profile reveals only the first few harmonics of the thicker scan compared to several harmonics at the thinner scan. Note, the first harmonic is rescaled to 1.06 rather than the usually assigned 1.0 in order to correct for the known low‐frequency enhancement of the in‐plane MTF from the high resolution kernel (filter) used for this scan.

Thus, by visual inspection of these Fourier Transform harmonics, because the same in‐plane resolution is used in both cases, one can gauge relative slice thickness effects.

### C. Effect of changing in‐plane resolution

**Figure 9:**
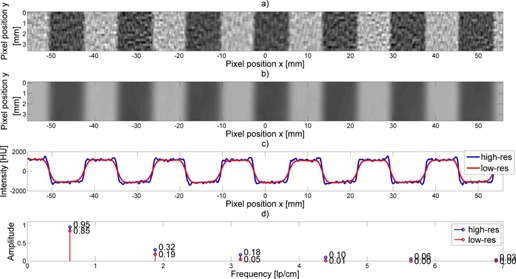
High‐resolution filter compared to low‐resolution filter. Waveform from a CT slice using (a) high‐resolution, (b) low‐resolution filter. A superimposition (c) of the extracted profiles. The resulting Fourier harmonics (d) for both profiles.

The Fourier Transform of the wave pattern encodes information about the in‐plane spatial resolution of the imaging device as described for example by the MTF. As is well known, a convolution (or blurring) due to resolution capabilities of the imaging device becomes a multiplicative filter (e.g., an MTF) in the spatial frequency domain. Thus, Fig. 9 portrays the difference between a waveform profile taken from a lower resolution filter versus a waveform profile taken from a higher resolution filter with the same slice width. Clear differences can be seen in the indicated amplitudes of the first several harmonics.

### D. Combined effects of x, y and z resolution

As the paper has tried to point out harmonic amplitudes of the waveform will reflect the combined effects of (x‐y) in‐plane resolution and z‐axis resolution (as scaled for ramp angulations) which combine to produce the 3D point spread function, psf (x, y, z).^(13)^

As a result, the amplitude of the harmonics in the Fourier Transform are influenced by the in‐plane modulation of Fourier amplitude as is characterized by the classical in‐plane MTF (which is a function of spatial frequency, $V_{x},V_{y}$) and the modulation and amplitude from the z‐axis MTF (which is a function of spatial frequency $V_{z}$). Moreover, the overall MTF can be calculated from analysis of the relative amplitude of the Wave harmonics, which will be reduced by the product of the in‐plane MTF and z‐axis MTF, appropriately scaled for ramp angle, at the corresponding harmonic frequency. This concept is illustrated in Fig. 9 which shows the agreement between the experimental amplitudes of the Wave Harmonics as appropriately modulated by the product of the in‐plane MTF and z‐axis MTF (scaled by ramp angle).

Likewise, the Wave Phantom provides a method to determine the MTF from the z‐axis slice thickness, presuming the in‐plane resolution is known and/or can be calculated from the six‐point sources (see Fig. 1(b)) which are provided with the Wave test pattern for point spread function and corresponding in‐plane MTF measurements. Similarly, in‐plane MTF resolution may be determined if the slice (thickness) sensitivity profile is known or can be measured (taking into account ramp angulation) because the overall MTF should be the multiplicative combination of in‐plane and z‐axis MTFs, as appropriately scaled for ramp angulation.

**Figure 10:**
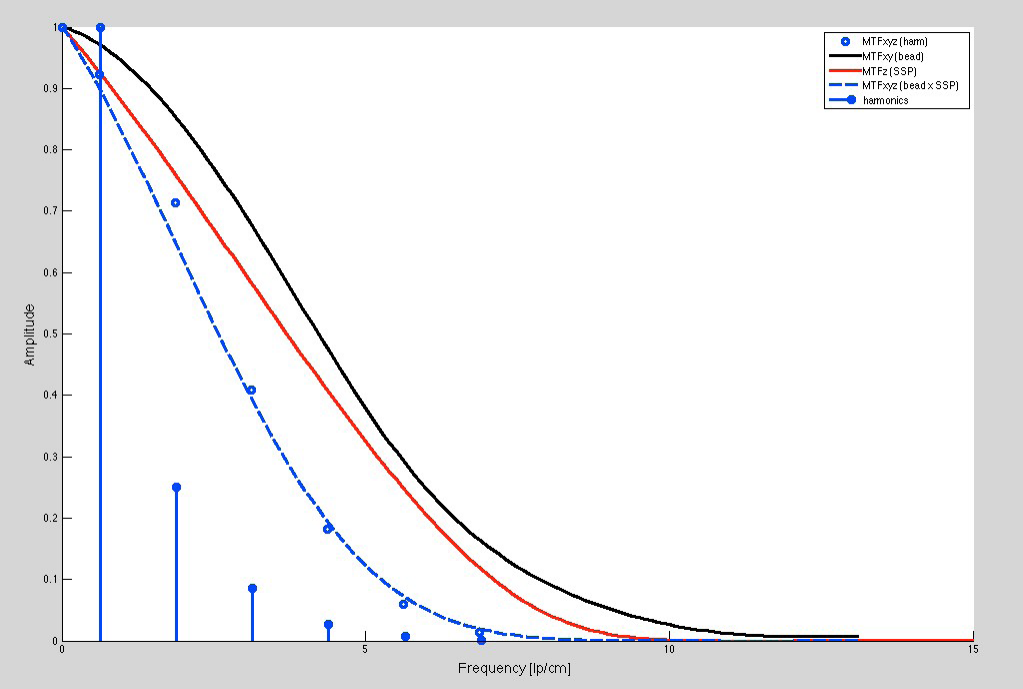
Comparison of ratio of measured to theoretical harmonic amplitudes and to those expected from overall MTF.

These interrelationships for the current Wave Pattern (30° ramp) regarding the overall MTF, in‐plane MTF, and z‐axis MTF are illustrated in Fig. 10.

As seen from Fig. 10, the in‐plane MTF from the transform of the point spread function is shown in black, along with the z‐axis MTF (appropriately scaled for the 30° ramp angle) (red), and their combined product yielding the overall MTF (dashed blue). It is used to reduce the amplitude of the fundamental harmonics at each appropriate harmonic frequency to yield the resulting amplitude of the harmonic, shown in open blue. The resulting points for the harmonic amplitudes show good agreement with those predicted from the overall MTF values at the corresponding frequency.

Thus, Fig. 10 shows that the resulting amplitude of the fundamental harmonics from the Wave Phantom do reflect the expected reduction amplitude by effect of the overall MTF resulting from the combined influence of in‐plane (x, y), and z‐axis MTF's. As such, the Wave Phantom provides visual (oscillating pattern) and quantitative information (harmonic amplitudes) on the combined effects of in‐plane and z‐axis imaging effects. Moreover, this is encoded in a single phantom rather than having separate measures of in‐plane and z‐axis resolution.

### E. Effect of body annulus and variation of position within the field of view of the scanned volume

A series of experiments were conducted to study the effect (if any) of the addition of a 30 cm body annulus to the Wave Phantom and to position the Wave Phantom both centered and off‐centered in the body annulus (11, 12).

In this way, variation of response in resolution resulting from BOTH x,y resolution and z‐axis (slice thickness) resolution can be studied. As a figure of merit for the combined effect, the amplitude for the third harmonic from the Fast Fourier Transform (FFT) of the Wave pattern is chosen. This choice results from the observation that the third harmonic is close to often perceived cutoff value of about 10% to 20% on a classical high‐resolution test pattern. The exact choice of harmonic would not significantly affect the trends reported in the experimental data shown below.

**Figure 11:**
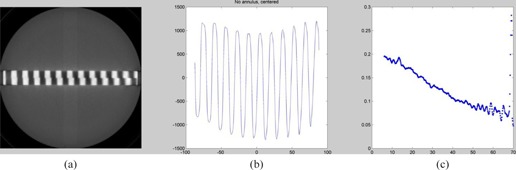
Scan of Wave module (a) in normal (20 cm) Catphan housing, without added annulus. Wave profile (b) of a function of distance (mm) of center of Wave. Third harmonic (c) from the local FFT as a function of distance (mm) from the center.

**Figure 12:**
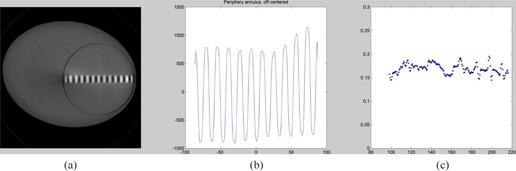
Scan of Wave module (a) in a 35 cm body annulus. Wave profile (b) as a function of distance (mm) of the Wave pattern. Third harmonic (c) from the local FFT as a function of distance (mm) across the wave when inserted in the 35 mm annulus.

Figure 11 illustrates how the third harmonic diminishes by more than 50% as one moves from the center of the field to the peripheral position. This finding probably results from both diminution in the spatial resolution (x,y) as one moves from the center of the field, as well as some typical slight broadening of the slice width profile due to the natural divergence of the X‐ray beam, as it moves from the tube to the detector.^(14)^

The results of Fig. 11 can be contrasted with Fig. 12 which shows the results from scanning the Wave phantom in a body mode with the addition of a 35 cm major axis elliptical annulus. One can note that the third harmonic amplitude of the Wave scanned in the body mode shows the maximum amplitude of the Wave pattern in Fig. 12(b) to be about 25% lower amplitude than the maximum amplitude of the same Wave phantom scanned in the head mode (Fig. 11(b)); however the third amplitude harmonic amplitude is relatively constant as one moves toward the periphery, probably because the slice width settings in the body mode have been designed to keep the slice width at the center of the field in the head vs. body mode, but the rate of divergence will be greater from the edge to the center of a 20 cm field of view, vs. from the edge to the center of a 35 cm field of view.

Thus, it appears that measures extracted from the Wave analysis, such as the third harmonic, may provide an interesting way to study spatial frequency response that results from combined in‐plane (x,y) and z‐axis resolution effects.

## IV. DISCUSSION/FUTURE WORK

The type of analysis suggested in the Materials and Methods section E on variation within the field of view will be extended to include the z‐axis location within the scanned volume, to investigate whether z‐axis variation due to divergence in multislice scanners and/or volume scanners can be usefully described with figures of merit, such as third harmonic amplitude.

The current results are based on 30° angled ramps that are used to augment z‐axis effects because of trigonometric magnification. However, ongoing work using 45° ramps avoids correction for 30° angulation and equally weights in‐plane and z‐axis effects.

The use of the Wave pattern will be further investigated as a tool for offering visual and analytic measures of 3D tomographic imaging devices, and may also offer a way to study nonuniformity effects throughout the CT plane (volume). The ideas of the Wave Phantom will be investigated for use with other modalities such as MRI, PET/CT, and SPECT/CT.

## V. CONCLUSIONS

The wave phantom offers a promising way to investigate 3D resolution as results from combined effect of in‐plane (x‐y) and z‐axis resolution, as contrasted to the use of simple 2D resolution gauges that need to be used with separate measures of z‐axis dependency, such as angled ramps. It offers both a visual pattern, as well as a pattern amenable to analytic analysis using Fourier Transform methods, and is closer to the diagnostic task where the 2D image has the hidden third (z) axis effects.

## COPYRIGHT

This work is licensed under a Creative Commons Attribution 3.0 Unported License.
